# Supplementary material for: Separable actions of acetylcholine and noradrenaline on neuronal ensemble formation in hippocampal CA3 circuits
Source: PLoS Comput Biol. 2021 Oct 1;17(10):e1009435. doi: 10.1371/journal.pcbi.1009435 (PMC8513881; doi:10.1371/journal.pcbi.1009435)
Supplement: S1 Fig — A-B) Latency, rise times, and jitter of mossy fiber driven EPSCs (n = 11) (A) and IPSCs (n = 12) (B). C-D) Reversal potential estimation of glutamatergic (n = 5) (C) and GABAergic (n = 6) (D) transmission at CA3 pyramidal cells. E-F) Spontaneous EPSC (n = 6) (E) and IPSC (n = 5) (F) frequency recorded before and after carbachol application. G-H) CA3 pyramidal cell input resistance (n = 5) (G) and holding current at -70 mV (n = 5) (H) before and after carbachol application. I) Dose-response of carbachol effect on IPSC amplitudes (n = 3). (PDF) [file pcbi.1009435.s001.pdf]

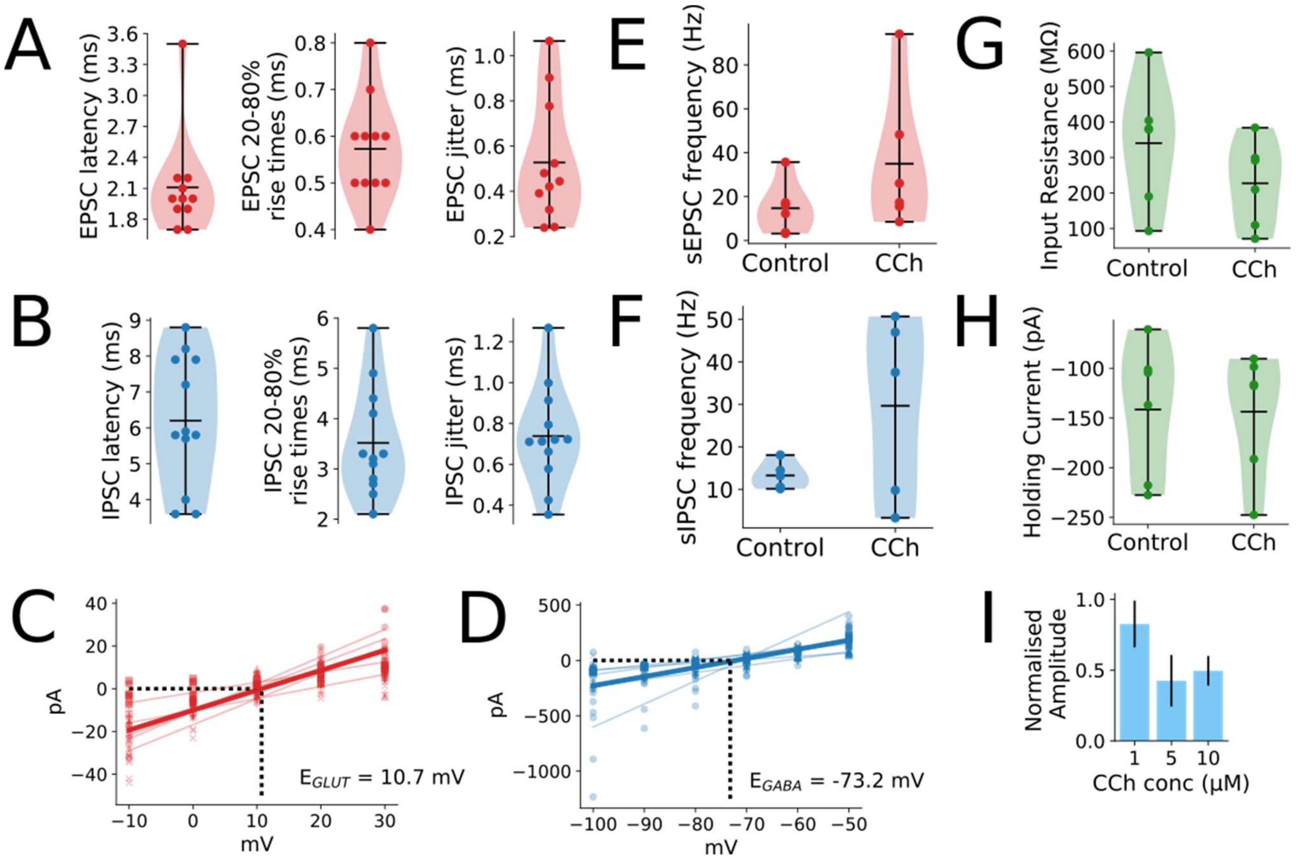

S1 Fig: A-B) Latency, rise times, and jitter of mossy fiber driven EPSCs (n = 11) (A) and IPSCs (n = 12) (B). C-D) Reversal potential estimation of glutamatergic (n = 5) (C) and GABAergic (n = 6) (D) transmission at CA3 pyramidal cells. E-F) Spontaneous EPSC (n = 6) (E) and IPSC (n = 5) (F) frequency recorded before and after carbachol application. G-H) CA3 pyramidal cell input resistance (n = 5) (G) and holding current at -70 mV (n = 5) (H) before and after carbachol application. I) Dose-response of carbachol effect on IPSC amplitudes (n = 3).
