# Supplementary material for: Separable actions of acetylcholine and noradrenaline on neuronal ensemble formation in hippocampal CA3 circuits
Source: PLoS Comput Biol. 2021 Oct 1;17(10):e1009435. doi: 10.1371/journal.pcbi.1009435 (PMC8513881; doi:10.1371/journal.pcbi.1009435)
Supplement: S2 Fig — A-B) Goodness-of-fit for EPSC (A) and IPSC (B) short-term plasticity models assessed by Bayesian posterior predictive p-values. Plots show the observed data for all experiments (EPSCs and IPSCs respectively) together with the expected values from the model before and after the application of 5 μM carbachol. p-values close to 0.5 indicate best fit. C-D) Posterior distributions for parameters of best fitting models given data for EPSCs (C) and IPSCs (D). (PDF) [file pcbi.1009435.s002.pdf]

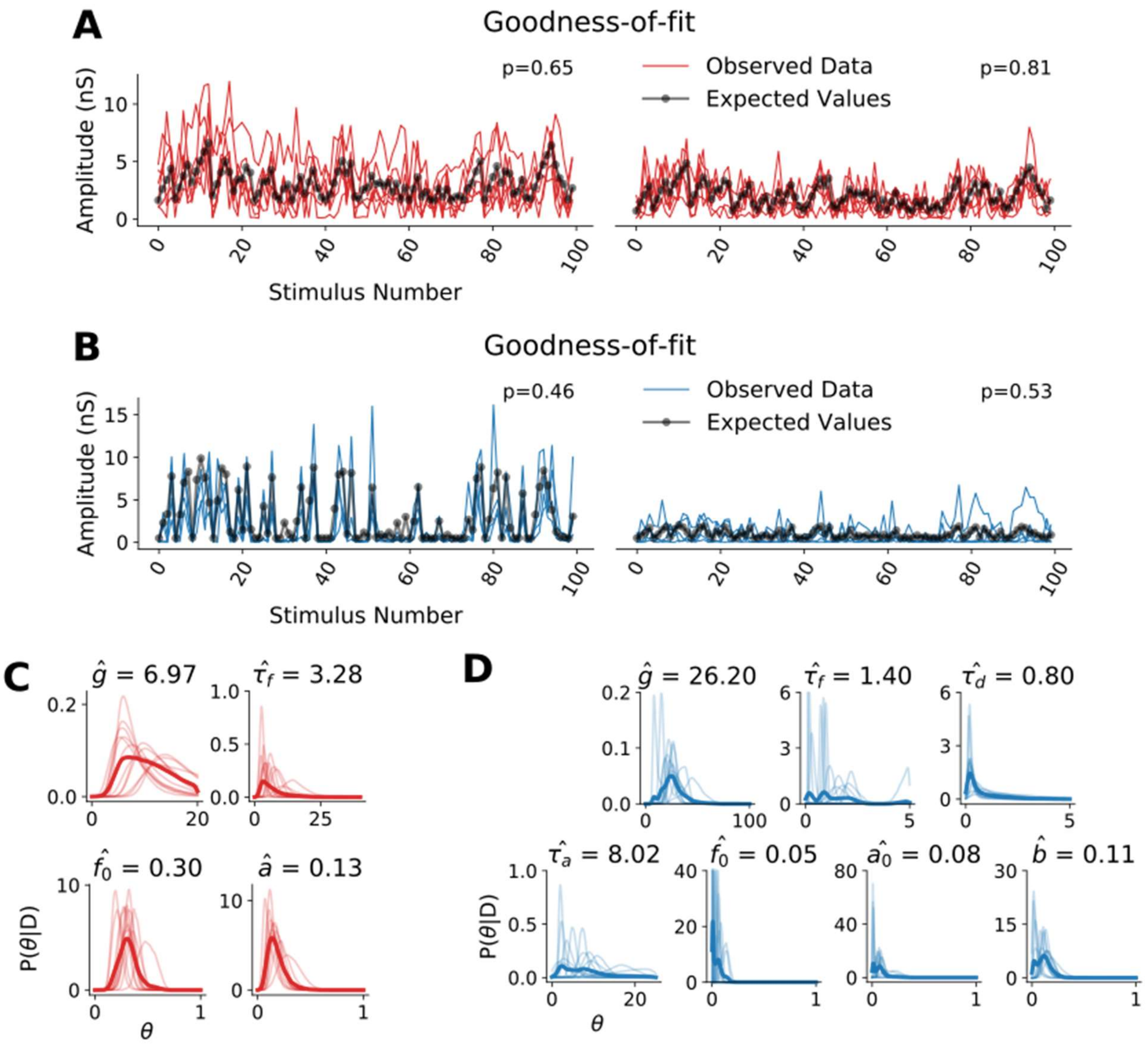

S2 Fig: A-B) Goodness-of-fit for EPSC (A) and IPSC (B) short-term plasticity models assessed by Bayesian posterior predictive *p*-values. Plots show the observed data for all experiments (EPSCs and IPSCs respectively) together with the expected values from the model before and after the application of 5 μM carbachol. *p*-values close to 0.5 indicate best fit. C-D) Posterior distributions for parameters of best fitting models given data for EPSCs (C) and IPSCs (D).
