# Supplementary material for: Separable actions of acetylcholine and noradrenaline on neuronal ensemble formation in hippocampal CA3 circuits
Source: PLoS Comput Biol. 2021 Oct 1;17(10):e1009435. doi: 10.1371/journal.pcbi.1009435 (PMC8513881; doi:10.1371/journal.pcbi.1009435)
Supplement: S3 Fig — A) Covariance in excitatory short-term plasticity model parameters (mean +/- sem). B) Covariance in inhibitory short-term plasticity model parameters (mean +/- sem). C) Example pairwise distributions of excitatory short-term plasticity model parameters D) Example pairwise distributions of inhibitory short-term plasticity parameters. (PDF) [file pcbi.1009435.s003.pdf]

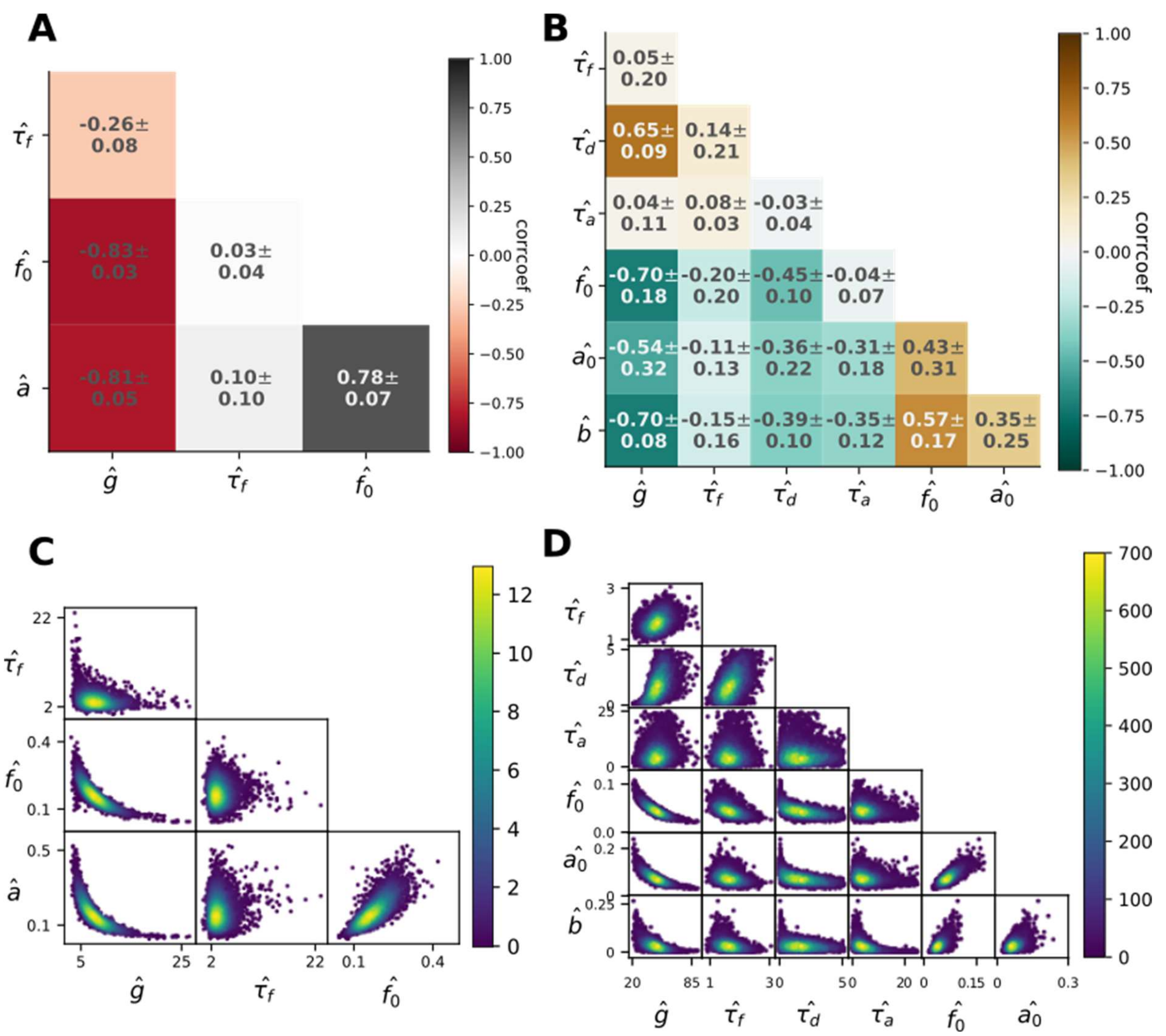

S3 Fig: A) Covariance in excitatory short-term plasticity model parameters (mean +/- sem). B) Covariance in inhibitory short-term plasticity model parameters (mean +/- sem). C) Example pairwise distributions of excitatory short-term plasticity model parameters D) Example pairwise distributions of inhibitory short-term plasticity parameters.
