# Supplementary material for: Separable actions of acetylcholine and noradrenaline on neuronal ensemble formation in hippocampal CA3 circuits
Source: PLoS Comput Biol. 2021 Oct 1;17(10):e1009435. doi: 10.1371/journal.pcbi.1009435 (PMC8513881; doi:10.1371/journal.pcbi.1009435)
Supplement: S4 Fig — A) All 15 cell morphologies plotted from NEURON spatial information. B) For each cell morphology, back-propagating action potential amplitude before (left) and after (middle) cholinergic modulation, and the difference in amplitude (right) are shown distributed across each CA3 pyramidal cell. (PDF) [file pcbi.1009435.s004.pdf]

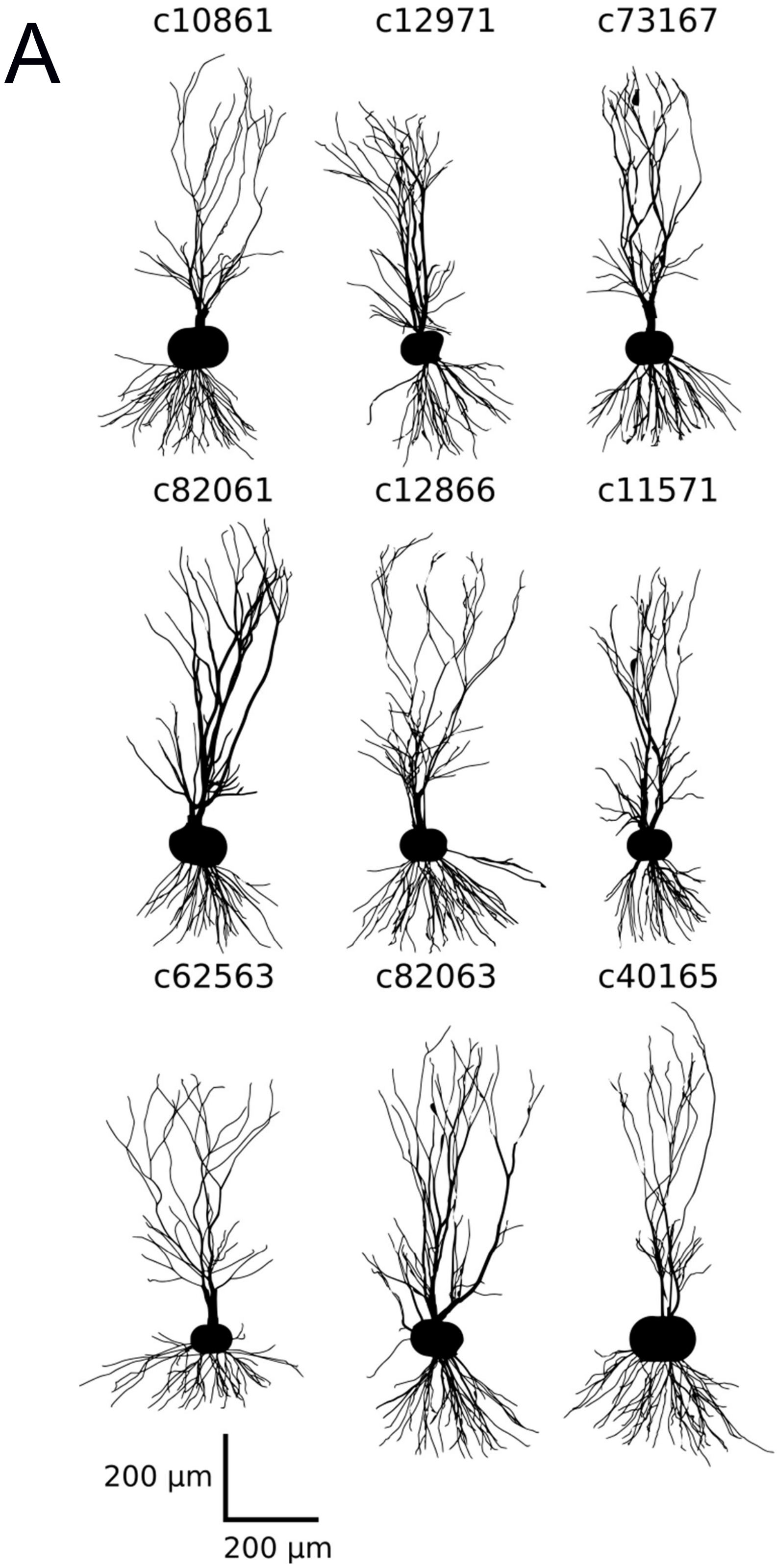
A
c10861
c12971
c73167
c20467
c31164
c82061
c12866
c11571
c60463
c73164
c62563
c82063
c40165
c81463
c60361
200 µm
200 µm

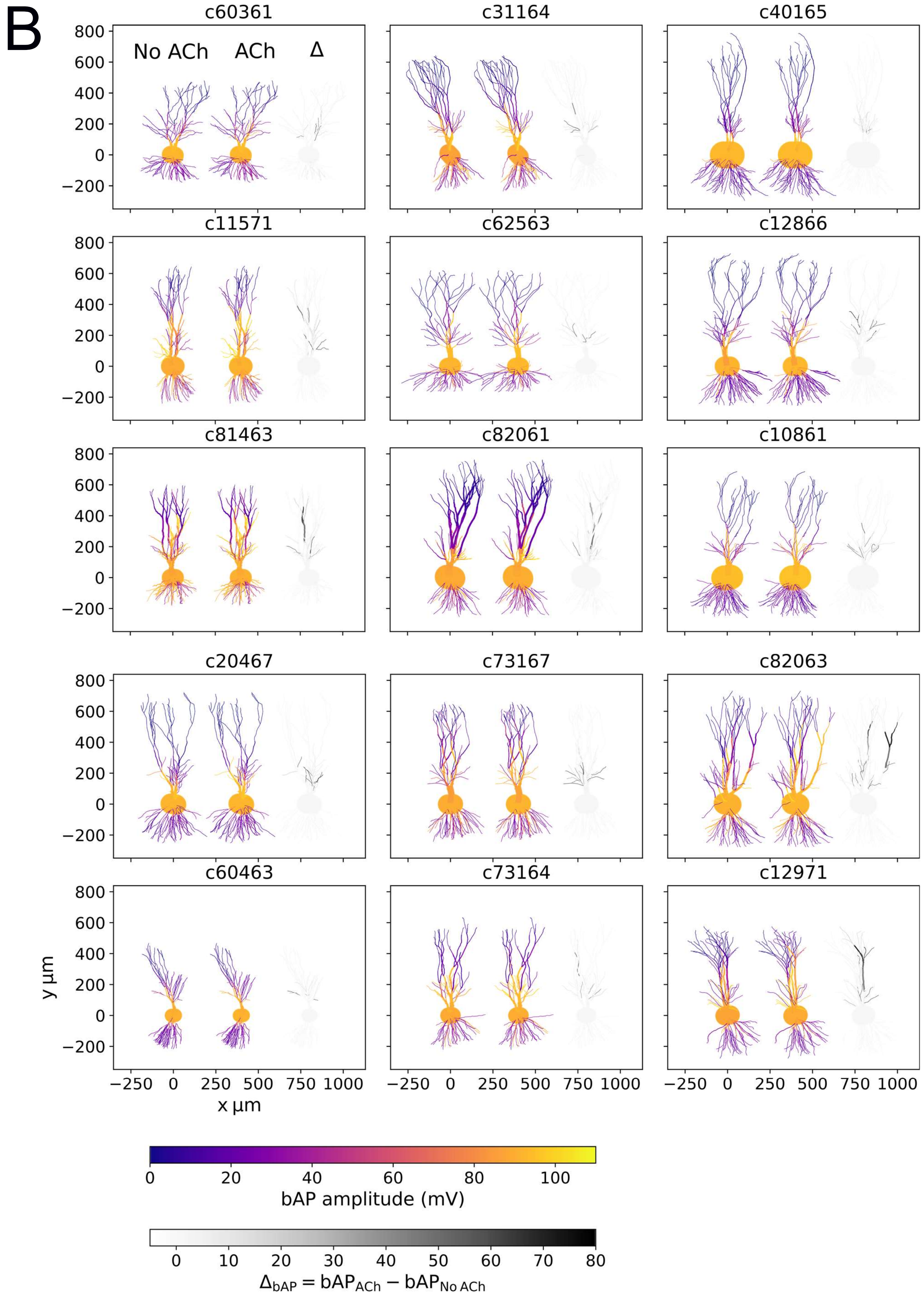
B
c60361
c31164
c40165
No ACh
ACh
Δ
c11571
c62563
c12866
c81463
c82061
c10861
c20467
c73167
c82063
c60463
c73164
c12971
800
600
400
200
0
−200
−250 0 250 500 750 1000
y µm
x µm
0 20 40 60 80 100
bAP amplitude (mV)
0 10 20 30 40 50 60 70 80
$\Delta_{bAP} = bAP_{ACh} - bAP_{No\,ACh}$

S4 Fig: CA3 pyramidal cell morphologies used for biophysical modelling. A) All 15 cell morphologies plotted from NEURON spatial information. B) For each cell morphology, back-propagating action potential amplitude before (left) and after (middle) cholinergic modulation, and the difference in amplitude (right) are shown distributed across each CA3 pyramidal cell.
