# Supplementary material for: Separable actions of acetylcholine and noradrenaline on neuronal ensemble formation in hippocampal CA3 circuits
Source: PLoS Comput Biol. 2021 Oct 1;17(10):e1009435. doi: 10.1371/journal.pcbi.1009435 (PMC8513881; doi:10.1371/journal.pcbi.1009435)
Supplement: S5 Fig — A) Slices of data shown in Fig 7C along frequency (top) and time (bottom) axes. B) Slices of data shown in Fig 8C along overlap (top) and time (bottom) axes. Colour coding for plots is indicated in the legend representing inclusion of different effects of acetylcholine in CA3. (PDF) [file pcbi.1009435.s005.pdf]

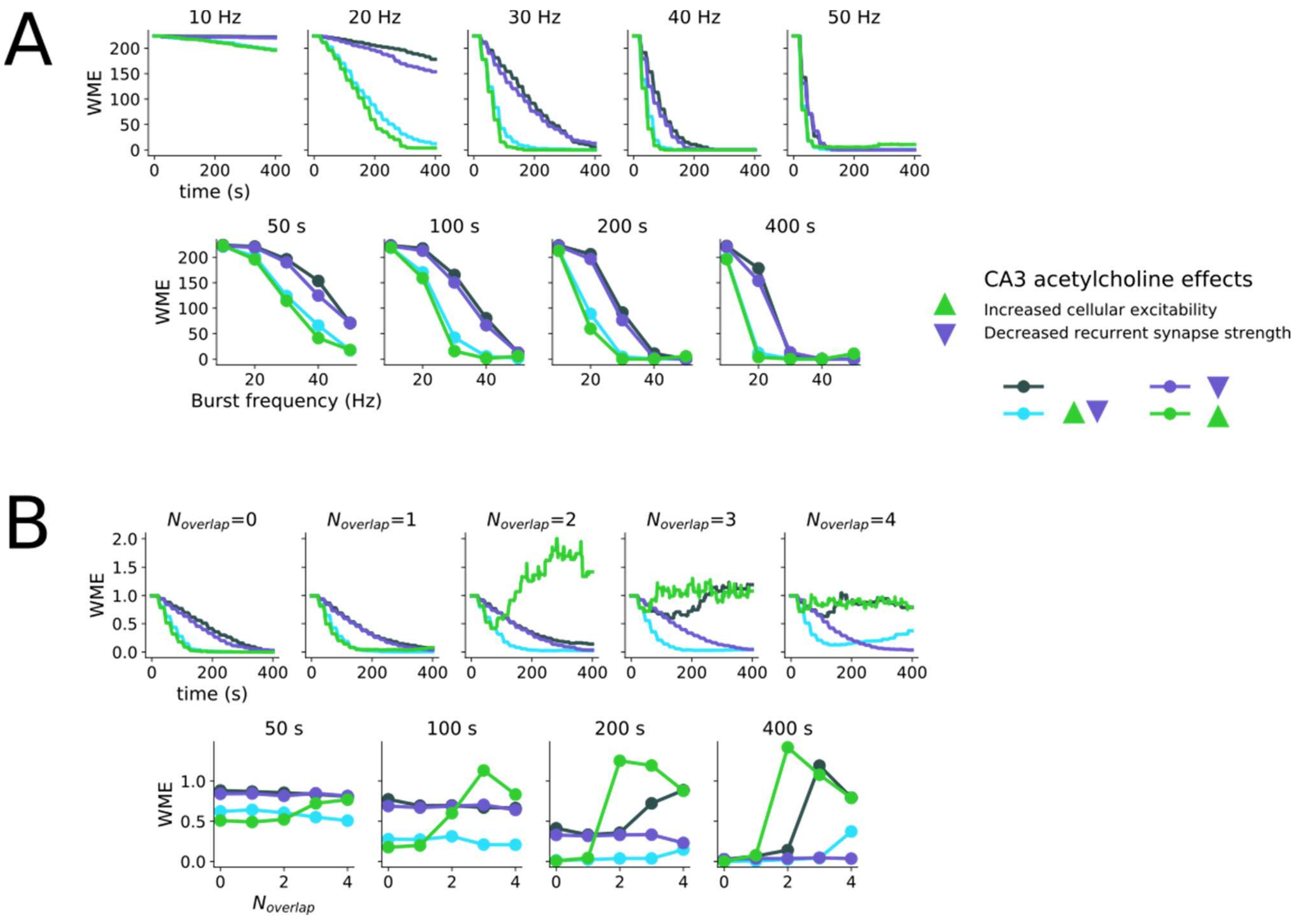

S5 Fig: A) Slices of data shown in Figure 7C along frequency (top) and time (bottom) axes. B) Slices of data shown in Figure 8C along overlap (top) and time (bottom) axes. Colour coding for plots is indicated in the legend representing inclusion of different effects of acetylcholine in CA3.
