# Supplementary material for: Separable actions of acetylcholine and noradrenaline on neuronal ensemble formation in hippocampal CA3 circuits
Source: PLoS Comput Biol. 2021 Oct 1;17(10):e1009435. doi: 10.1371/journal.pcbi.1009435 (PMC8513881; doi:10.1371/journal.pcbi.1009435)
Supplement: S6 Fig — Model CA3 spiking activity (left) and resulting pyramidal cell weight matrix (right) with mossy fiber bursting at 30 Hz (A), 20 Hz (B), and 20 Hz with cholinergic modulation (C). (PDF) [file pcbi.1009435.s006.pdf]

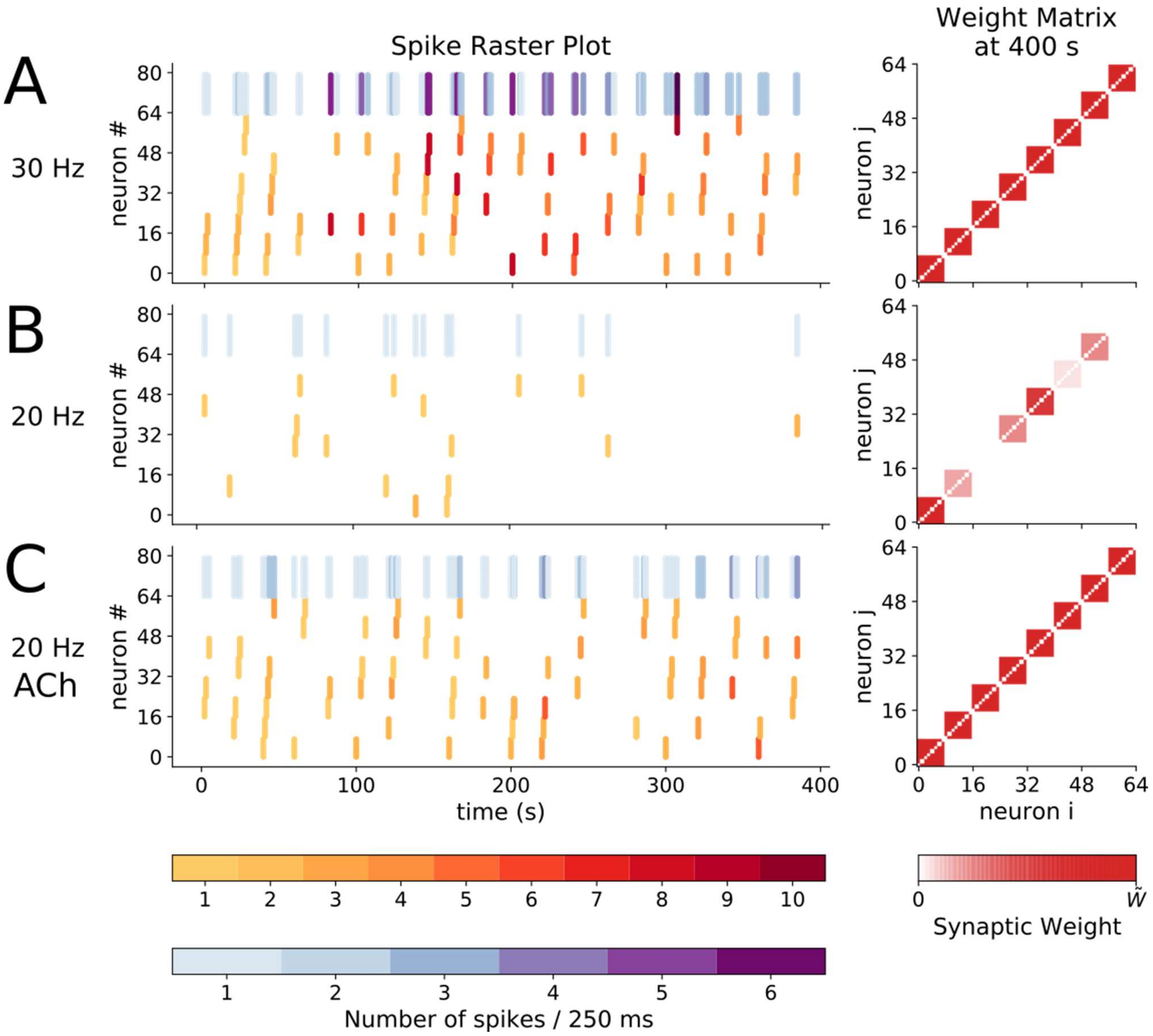

S6 Fig: Model CA3 spiking activity (left) and resulting pyramidal cell weight matrix (right) with mossy fibre bursting at 30 Hz (A), 20 Hz (B), and 20 Hz with cholinergic modulation (C).
