# Supplementary material for: Separable actions of acetylcholine and noradrenaline on neuronal ensemble formation in hippocampal CA3 circuits
Source: PLoS Comput Biol. 2021 Oct 1;17(10):e1009435. doi: 10.1371/journal.pcbi.1009435 (PMC8513881; doi:10.1371/journal.pcbi.1009435)
Supplement: S7 Fig — Model CA3 spiking activity and resulting weight matrix without cholinergic modulation (A) and with cholinergic modulation (B) with 0 (top), 2 (middle), and 4 (top) cells overlapping between ensembles. (PDF) [file pcbi.1009435.s007.pdf]

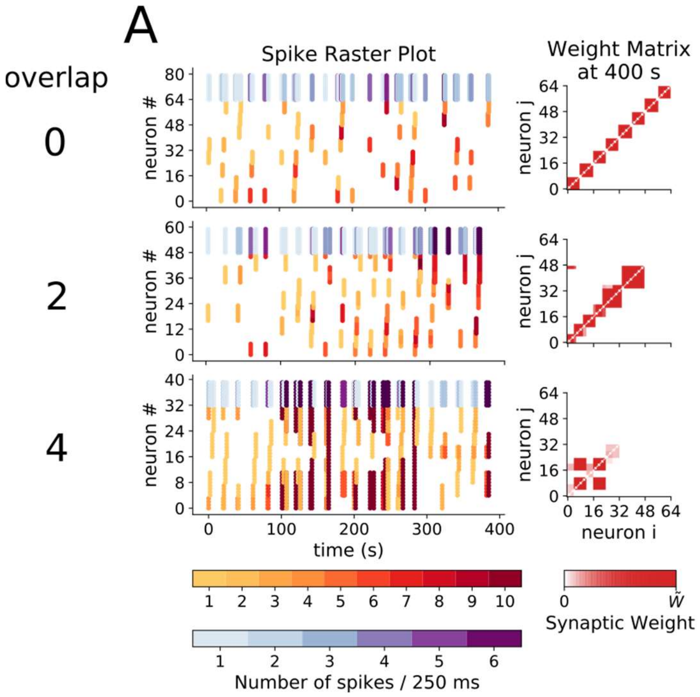

S7 Fig: Model CA3 spiking activity and resulting weight matrix without cholinergic modulation (A) and with cholinergic modulation (B) with 0 (top), 2 (middle), and 4 (top) cells overlapping between ensembles.
