# Supplementary material for: Separable actions of acetylcholine and noradrenaline on neuronal ensemble formation in hippocampal CA3 circuits
Source: PLoS Comput Biol. 2021 Oct 1;17(10):e1009435. doi: 10.1371/journal.pcbi.1009435 (PMC8513881; doi:10.1371/journal.pcbi.1009435)
Supplement: Attachment — Submitted filename: review.pdf [file pcbi.1009435.s008.pdf]

Prince et al describe a combined experimental/theoretical approach on how acetylcholine (ACh) and noradrenaline (NA) affect hippocampal CA3 circuits. They focus on input from dentate gyrus, which is important to control firing of CA3 pyramidal cells.

Acetylcholine is proposed as a gatekeeper for encoding of memories in CA3 (very much in line what was proposed by Hasselmo and others for ACh mediated changes between wake and sleep), and noradrenaline is proposed to have more limited effects. The main effect found (in experiments) for acetylcholine is the suppression of feed-forward inhibition (especially after a few pulses in a burst stimulation) in the mossy fiber pathway to the CA3 network. This suppression was interpreted to be mediated (Fig. 2F) by a reduction in inhibitory postsynaptic conductance (partially counteracted by an increase of baseline facilitation). In contrast, NA was found to increase mainly the time constant of recovery from synaptic depression.

Prince et al also showed that feedforward excitation in the mossy fiber pathway was suppressed by CCh (Fig 1C), and this suppression was interpreted to be mediated mostly by a reduction to about 50% of postsynaptic conductance (Fig.2E).

CCh was also found to suppress recurrent synaptic connections (Fig. 6C) and to increase excitability of CA3 pyramidal neurons (Fig. 6D), which confirms previous experimental work by others; NA had no significant effect.

MAJOR ISSUES

In Fig. 1C (see also Fig. 6A), why was there no recovery after washout of CCh? Please explain in the manuscript why there was no recovery to 100%. I found this lack of recovery for EPSCs surprising also because IPSC amplitudes did recover. Is the depression of EPSCs really ``irreversible'' (line 203)? How would such an irreversibilty be interpreted functionally?

Why were the results after washout of NA not investigated/shown (Fig. 1E,F)? In Fig. 6B, EPSC amplitude seems to decrease in the recovery period.

I found the results on drug-induced changes of parameters of short term plasticity of inhibition unconvincing because the error bars (SEMs) in Fig. 2F are huge. Furthermore, the chosen model framework for short-term plasticity suffers from the fact that very different combinations of parameters can lead to very similar results (degeneracy and correlation between parameters). Therefore I have some doubts about the differential effects of CCh and NA on parameters. Because such differential effects are key aspects of the manuscript, they might deserve more attention. It is tempting to speculate that many more parameters of the short-term plasticity model would have changed significantly if more experiments would have been performed. This uncertainty about the true effects of the drugs on short-term plasticity dampens my enthusiasm about their functional interpretation in later parts of the manuscript.

In the numerical simulations of the manuscript (e.g. Figs 3, 4) specific sets of parameters for short-term plasticity were used, without taking into account their uncertainty. It remains unclear (at

least to me) which parameter changes in the end controls the CCh induced change of the E-I ratio in Fig. 3E or back-propagating APs in Fig. 4.

Even though CCh strongly reduced inhibition, the simulated effects of the drug on E-I ratio (Fig. 3E) appear to me rather small. There is a an effect on the E-I ratio in Fig. 3E, in which the red region became slightly larger, but there is no fundamental change in the overall picture. Also the effects on back-propagating APs in Fig. 4CDE are not that striking, even though few dendrites may show a strong effect (Fig. 4B,C), which then could affect plasticity.

I found that the two sentences on line 436 exaggerate the effects of CCh and NA: ``Our simulations indicate that disinhibition of the feed-forward mossy fiber pathway by acetylcholine or noradrenaline is a requirement for reliable induction of synaptic plasticity between CA3 pyramidal cells when CA3 ensembles are activated by mossy fiber inputs. Furthermore, acetylcholine reduces the number of stimuli within a burst required to generate substantial dendritic calcium whereas noradrenaline only facilitated the back-propagation of action potentials once they were initiated and is therefore only important for calcium signalling driven by longer bursts with more stimuli.'' Again, there are strong effects in some examples dendrites (Fig. 4B), but on average the effect is seems to be weak (Fig. 4 DE).

Starting with Figure 5, the manuscript becomes more speculative and merely illustrates various scenarios related to synaptic plasticity. Now, very different kinds of numerical simulations of different sets of models are used, and these models depend on multiple (often unconstrained, ad-hoc chosen) parameters. Figs. 5, 7, and 8 deal with fully connected networks of excitatory ind inhibitory point neurons, spike timing dependent plasticity rules including scaling rules ``to maintain constant overall spiking dynamics'', and short-term plasticity models as in Fig. 2. However, the firing statistics are now different from what was used earlier.

In the simulations in Fig. 5, mossy fiber input was compared to perforant path input. But very specific assumptions on firing rates of PP inputs (line 479) were made (based on data from the pre grid-cell era). Findings in Fig. 5 probably depend critically on many details of the model setup and the chosen parameters, which was not investigated. Yet another firing statistics for mossy fibers was used in Fig. 5C (winner take all process with adaptation). What was the motivation for this set of simulations? Overall, Fig. 5C (text, figure, and caption) was puzzling to me, and I think Fig. 5C is not well enough connected to other parts of the manuscript.

Fig. 6 illustrates an experimental result. Prince et al. confirmed earlier results from other groups: CCh caused a ~50% depression of recurrent CA3-CA3 excitatory synaptic transmission and there is an increase in paired pulse ratio (PPR). Furthermore, CCh increased input resistance (but this result seems to be not significant: see Fig. 6D, right) and depolarized CA3 pyramidal cells. New (to my knowledge) is that NA had no (significant) effect on CA3 neurons and the recurrent synapses.

In Fig. 6, are the stimulations used to evoke responses also ``minimal'' as for the mossy fibers? Or are the shown responses the sum of inputs from many active synapses? The EPSC amplitudes (200 pA in Fig. 6A) are comparable in magnitude to mossy fiber EPSCs in

Fig. 1, even though I thought mossy fiber synapses are much stronger than recurrent synapses in CA3. Please clarify.

Figure 7 illustrates with simulations ensemble formation in CA3. For the simulations in Fig. 7, NA was assumed to ``enable synaptic plasticity between CA3 pyramidal cells'' (line 575). Please add here the citations for experimental evidence on this. Was in Fig. 7 CCh assumed to NOT enable synaptic plasticity? Or was plasticity assumed to be the same for NA and CCh? I think this point should be made very clear and explicit here. This is important to be able to interpret the statement in line 589: ``Remarkably, when noradrenaline was switched for acetylcholine by applying the CA3 network effects, ensemble formation was rescued at the lower burst frequency.'' I find this surprising because earlier NA was proposed to control plasticity of these synapses. So, did the replacement of NA by CCh change (reduce) the plasticity of synapses? If yes, how was this implemented?

For the simulations shown in Fig. 7, to mimic the effects of CCh, the resting membrane potential for excitatory cells was depolarized by 5 mV to -70 mV, which was motivated by experiments (Fig. 6D). However, ``inhibitory cells were also depolarized to -63 mV'' (line 573). Why is this necessary? Is there experimental evidence on this effect?

Fig. 7B: How long were the simulations? (for comparison with Fig. 7C)

The main finding of Fig. 7 is summarized here ``...key factor [for ensemble formation] was the increase in cellular excitability, as removing the parameter changes to cellular excitability abolished the effect of acetylcholine but removing reductions in CA3-CA3 recurrent connections did not'' (line 602). Why were the changes induced by acetylcholine on the STP model parameters not important? Was this even investigated in Fig. 7? That these question were not addressed indicates to me a lack of coherence of the manuscript.

I found the result in Fig. 7 not surprising at all given the fact that recurrent synapses (maximum conductance 0.5 nS in line 1077) should be much weaker than feedforward mossy fiber synapses, even though I could not find in the manuscript a number on the maximum conductance g of mossy fibers. My rough estimate from Fig. 1 (with current amplitudes in the range of 500 pA or more) and from the Supplementary Fig. 1 (assuming a driving force for excitatory currents in the range of 100 mV) is that maximum conductances of 500 pA / 100 mV = 5nS are easily achieved, and g should be even higher. The weak recurrent synapses are of minor importance for ensemble formation simply because they are weak.

More interesting in this respect are the results in Fig. 8: A reduction in strength of the (weak) CA3-CA3 recurrent synapse mainly determines the ability for acetylcholine to allow greater ensemble overlap. However I think this insight is not really new, and already Hasselmo 1999 explained in great detail that acetylcholine is could be involved in ``partially suppressing excitatory feedback connections and so facilitating encoding without interference from previously stored information''.

In summary, in the simulations shown by Prince et al. the huge parameter space was not explored. Often, many specific parameter

values were chosen arbitrarily, I think. The theory is therefore more like an illustration of how it could be rather than a framework from which solid, experimental testable predictions could be derived, and how these predictions quantitatively would depend on parameters.

A limitation of the approach by Prince et al. is that they neglect the putative neuromodulation of direct inputs to dendrites of CA3 cells from entorhinal cortex (even though this perforant path was considered in simulations in Fig. 5). They fully neglect feedback from CA3 to DG via mossy cells, which might modulate granule cell activity for repeated stimulations. The authors could better justify why these features of hippocampal circuits are neglected.

MINOR ISSUES

line 251: delete extra ``a'' before ``produced''

line 317: replace ``increase in p0'' by ``increase in f0'' ?

line 323: ``increase in the facilitation incremental parameter tau_d.'' Do you mean here the ``depression incremental parameter''? Moreover, the text does not match results shown in Fig. 2F in which NA was indicated to significantly change only tau_a

Fig. 3B,C. the EPSCs are plotted too small to be able to evaluate short-term plasticity. Please use a different current scale bar for EPSCs (maybe 100pA or lower).

Caption of Fig. 3 describes ``Pulse ratios for 2nd, 4th and 6th pulses'' whereas panels D and E figure shows ``n=2, n=6, n=10''

Caption of Fig. 5C: ``50 feed-forward inputs to a single granule cell'' . Do you mean ``... single pyramidal cell'' instead?

line 1079: I could not find the announced ``Table 1'' on network parameters
