# Supplementary material for: Separable actions of acetylcholine and noradrenaline on neuronal ensemble formation in hippocampal CA3 circuits
Source: PLoS Comput Biol. 2021 Oct 1;17(10):e1009435. doi: 10.1371/journal.pcbi.1009435 (PMC8513881; doi:10.1371/journal.pcbi.1009435)
Supplement: Attachment — Submitted filename: Response to reviewers PLoS Comp Biol 21.03.24.docx [file pcbi.1009435.s009.docx]

Response to reviewers’ comments (comments in black, responses in blue)

**Reviewer #1:**

It has long been believed that neuromodulation acts as a gatekeeper of the hippocampal memory system, deciding which aspects of experience are novel/important enough to warrant memory storage. The mechanisms of this process are however not completely well understood: for instance both acetylcholine and noradrenaline can affect the input into the system (via the DG-CA3 mossy fiber connection), but their relative contribution to memory gating is unclear. This manuscript uses slice electrophysiology to jointly characterize the role of acetylcholine (Ach) and noradrenaline (NA) on the mossy fiber excitatory input and di-synaptic feedforward inhibition. These findings are incorporated into computational models of ensemble formation to gauge their functional implications.

ACH was shown to induce a lasting reduction in the amplitude of mossy fiber EPSPs. It also caused a reduction of IPSPs, while also reducing facilitation ratios. Thirdly, it increased the excitability of CA3 cells. For NA, the applied dose had no effect on EPSCs and a weaker effect of IPSCs. Careful model comparison also revealed differences w.r.t. short term plasticity. Ach lead to much bigger shifts in EI balance than NA, with potentially important implications to CA3 recurrent synapses plasticity, as evidenced by compartmental models.

These processes are then incorporated into a spiking recurrent network to investigate ensemble formation, where ensembles here are defined somewhat extremely (c.f. lines 469-70) as fully connected components completely decoupled from the rest of the network. This model is uses to iteratively investigate the contribution of different components (mostly numerically)

Major:
While the numerical results are clear, there seem to be a lot of knobs in the modeling approach (and to some degree the experiment), which make it hard to infer the generality of the conclusions; I would have appreciated a bit more more explanation on how closely does the modeling setup reflect known physiology, and how robust the results are to changing different parameters.

We are happy to provide further clarification on this important point. All of the models are based on physiological parameters wherever possible. The Tsodyks-Markram STP model used in Figure 2 is commonly used to describe STP and is based on the known mechanisms underpinning release of vesicles from presynaptic terminals and their variation with frequency of activation. The biophysical model used in Figure 4 is based on physiological parameters taken from Henze et al 1996 and Hemond et al 2008 that set CA3 neurons to respond to current with burst firing properties as shown in Traub 1989 and Pinsky and Rinzel (1994). The CA3 network model used in Figures 5, 7 & 8 uses parameters taken from Hummus et al 2014 which in turn used physiological data from Brown and Randall (2009) for CA3 pyramidal cells and Buhl et al (1996) for inhibitory basket cells. In all instances we have not changed the parameters from those reported in physiological experiments unless to test the effects of acetylcholine or noradrenaline. We have now adjusted the text in a number of places to indicate the sources and rationale for parameterisation (lines 162, 255, 403, 424, 427-434, 487, 493-498, 513, 519, 615, 617, 621-627, 1094, 1123, 1125, 1147).

We have also tested the robustness of the results to changing parameters in a number of instances. Firstly, we have now performed a detailed analysis of the covariance of parameter estimation for the effects of acetylcholine and noradrenaline on STP in the Tsodyks-Markram model (see new Supplementary Figure 3). This analysis shows that, as expected, there is a strong correlation between some parameters indicating a number of possible parameter sets that could explain the effects of acetylcholine and noradrenaline. However, when running our Markov Chain Monte Carlo (MCMC) inference procedure we chose priors that bias our results to keep these parameter values small since these are likely to be most physiological. As a result, despite correlation amongst parameter values, we are confident that the inferred parameters are in a realistic regime, and the effects observed by introducing cholinergic and noradrenergic agonists reflect changes within this regime. Therefore, we are confident that the results we present are robust to varying parameterisation when constrained to physiologically relevant values. In addition, we have specifically used naturalistic patterns of activity in mossy fibre axons to constrain the STP model in order to be able to generalise across all activity patterns (Figure 2). In Figure 3 we sample a wide range of activity patterns to gauge the impact of changing E-I balance and find that the effects of acetylcholine and noradrenaline are strongest in the physiological range indicated within our naturalistic activity patterns. For the biophysical model in Figure 4, we have used a set of 15 separate pyramidal neuron morphologies to demonstrate generalisability across neurons and tested the effects of changing E-I balance across a full range of values to show the range where changing E-I balance has strong effects. Finally, in Figures 7 and 8 (and supplementary Figures 5, 6 & 7) we show the effects of acetylcholine and noradrenaline across a range of overlap sizes and burst frequencies again demonstrating the generalisability of the result.

The order in which the information is presented is a bit odd in places, with results being anticipated and then repeated in multiple sections and going back and forth between experiments and models, making the individual points not as sharp as they could be, see also detailed comments below. A more effective narrative thread would be to 1) identify all the different immediate effects of different neuromodulators, then 2) put them together in a circuit model to predict differences in ensemble formation, and lastly 3) take the different effects apart to reveal which consequence of neuromodulation has most effect functionally.

We appreciate the point made by the reviewer, but it is important to highlight the nature of the study is necessarily in 2 parts that are linked sequentially. In the first section we found that acetylcholine and noradrenaline had differential effects on mossy fibre transmission to CA3 and in particular the regulation of feedforward inhibition. Therefore, we first had to investigate the implications of this result on plasticity within the CA3 network before we could proceed to investigate the impact of neuromodulation on ensemble formation in the CA3 network. We do not feel it would make sense to show the impact of neuromodulation on the CA3 network before we had ascertained that it could facilitate synaptic plasticity triggered by mossy fibre inputs. In view of this logical sequence, we prefer to keep the organisation of the manuscript as it is.

I would have liked some concrete predictions coming from the model; for instance w.r.t. effects of systemic neuromodulator manipulation on memory ability of animals — I would think that the generalization ability for different memories may also be affected (going with the allowable degree of overlap across ensembles).

There are many and various predictions that might be made from our results and some of these are discussed in the section of the discussion lines 830-859. We have made further additions to these along the lines suggested by the reviewer relating to the recent paper from Susumu Tonegawa’s group (Sun et al Nat Neurosci 2020) indicating that CA1 place cells provide a readout of generalised ensemble inputs from CA3. Our results predict that the formation of such event codes depends on acetylcholine but not noradrenaline release.

Minor:

Somehow confounding how the change in E(I)PSC amplitude and depression/facilitation are talked about w.r.t to fig 1 and 2. The title suggests that short term plasticity should be referred to in fig 2 and associated text but is already mentioned w.r.t. fig 1. Overall, some of the text felt repetitive. There may be a better way to streamline the presentation of the two...

We agree that the flow of text was not optimal here. We have now altered the subheading for Figure 1 results to include the results in Figure 2 and deleted the subheading for Figure 2. The single subheading for the results presented in Figures 1 and 2 now reads “*Acetylcholine and noradrenaline reduce feed-forward inhibition and alter short-term plasticity in the mossy fiber pathway”.*

don’t get the logic of the statement in line 219: the EI ratio changes seem quite dramatically different between Ach and NA manipulations even at the raw data level, why say they could be similar? In fact the results contradict it.

Although the results for the separate neuromodulation of EPSCs and IPSCs for acetylcholine and noradrenaline are very different, because the effect of acetylcholine is greater for both EPSCs and IPSCs it is not obvious from these data that the *overall E-I ratio* is altered. And specifically, due to the dependence of the effects on stimulation frequency, it is unclear under what stimulation patterns the E-I ratio will be altered. That is the logic behind the statement and why we needed to test this directly.

Line 330-331: The effects in inhibition seem contradictory: high firing rates and strong synaptic depression seems like a particularly energy inefficient way of neurally implementing a release from inhibition. Rather it would indicate a shift towards different patterns of spike timing…

We agree that a dual mechanism for cholinergic modulation of inhibition where spike rate is increased but transmission depressed appears counter intuitive. However, these are the results that we observe and therefore the ones that we use to model the effects on driving the CA3 network. There may be a slight shift to shorter latency spike responses in the interneurons driven by mossy fiber input, but it is not clear that this would have much effect on our results particularly when the overall effect on is such a strong dis-inhibition.

Line 495: “a colored noise signal” feels odd; maybe just ‘colored noise’ (?)

We have revised the text to “colored noise input”.

L536: the role of Ach on excitability has been mentioned in the results before, rather apologetically, leaving the reader to wonder whether it was due to simply a release in inhibition or also involved changes in intrinsic neural properties. Now we go back to experiments and measure that in the middle of setting up possible coding properties of the Ach manipulation; I feel the narrative would be better served if the experimental results were more coherently explained and closer together in the storyline.

This refers to the comment above about the logical sequence of the manuscript. For the reasons given above we prefer to retain the organisation of the manuscript as it is. We think it would be hard to provide a rationale for the experiments in Figure 6 before we ascertained a role for neuromodulation in enhancing plasticity in the recurrent CA3 network.

L590: “applying the CA3 network effects” phrasing odd

We have adjusted this phrase to “applying the observed effects of acetylcholine on the CA3 network”.

The link to neuromodulators signaling different forms of uncertainty is out of place with the rest of the ensemble formation discussion, I’d try to integrate it better.

We have reworked this final section of the discussion and we hope these ideas are now better integrated. The theories for separable roles of noradrenaline and acetylcholine in uncertainty are highly influential and one of the few instances where comparisons have previously been made between the two neuromodulators so we feel it is important to include.

**Reviewer #2:**

Prince et al describe a combined experimental/theoretical approach on how acetylcholine (ACh) and noradrenaline (NA) affect hippocampal CA3 circuits. They focus on input from dentate gyrus, which is important to control firing of CA3 pyramidal cells.

Acetylcholine is proposed as a gatekeeper for encoding of memories in CA3 (very much in line what was proposed by Hasselmo and others for ACh mediated changes between wake and sleep), and noradrenaline is proposed to have more limited effects. The main effect found (in experiments) for acetylcholine is the suppression of feed-forward inhibition (especially after a few pulses in a burst stimulation) in the mossy fiber pathway to the CA3 network. This suppression was interpreted to be mediated (Fig. 2F) by a reduction in inhibitory postsynaptic conductance (partially counteracted by an increase of baseline facilitation). In contrast, NA was found to increase mainly the time constant of recovery from synaptic depression.

Prince et al also showed that feedforward excitation in the mossy fiber pathway was suppressed by CCh (Fig 1C), and this suppression was interpreted to be mediated mostly by a reduction to about 50% of postsynaptic conductance (Fig.2E).

CCh was also found to suppress recurrent synaptic connections (Fig. 6C) and to increase excitability of CA3 pyramidal neurons (Fig. 6D), which confirms previous experimental work by others; NA had no significant effect.

MAJOR ISSUES

In Fig. 1C (see also Fig. 6A), why was there no recovery after washout of CCh? Please explain in the manuscript why there was no recovery to 100%. I found this lack of recovery for EPSCs surprising also because IPSC amplitudes did recover. Is the depression of EPSCs really ``irreversible'' (line 203)? How would such an irreversibilty be interpreted functionally?

Prolonged applications of CCh have been shown to cause long-term depression of excitatory transmission by removal of AMPA receptors from the synaptic membrane (Dickinson et al., Mol Brain 2009). It is entirely feasible that this mechanism is acting in our experiments although we have not explored this directly. It is also possible that the transmission eventually recovers over longer timescales beyond the frame of our experiments. We have not explored the implications of long-term depression beyond the period of CCh application since our results concern the impact of acetylcholine or noradrenaline during exposure to these neuromodulators and not afterwards. We now include reference to the Dickinson paper in the text (line 186-187) and have removed the word irreversible since, as the reviewer points out, we cannot be sure of this.

Why were the results after washout of NA not investigated/shown (Fig. 1E,F)? In Fig. 6B, EPSC amplitude seems to decrease in the recovery period.

The lack of a washout period in Figure 1 is an omission on our part. Noradrenaline did not have any effect on EPSCs so we did not include a washout period and we kept the experimental protocol the same for IPSCs. As mentioned above, the washout period is not critical for our study so we do not believe this detracts from our findings.

There is some normal biological variability in the EPSC amplitudes during and after noradrenaline application in Figure 6 but no significant changes were observed.

I found the results on drug-induced changes of parameters of short term plasticity of inhibition unconvincing because the error bars (SEMs) in Fig. 2F are huge. Furthermore, the chosen model framework for short-term plasticity suffers from the fact that very different combinations of parameters can lead to very similar results (degeneracy and correlation between parameters). Therefore I have some doubts about the differential effects of CCh and NA on parameters. Because such differential effects are key aspects of the manuscript, they might deserve more attention. It is tempting to speculate that many more parameters of the short-term plasticity model would have changed significantly if more experiments would have been performed. This uncertainty about the true effects of the drugs on short-term plasticity dampens my enthusiasm about their functional interpretation in later parts of the manuscript.

We apologise that we omitted to specify the error bars correctly. The error bars are SD not SEMs. We have now clarified this in the text (lines 312, 316) and the figure legend. Therefore, the variability in the results is much smaller than surmised from the data as first presented. We share the reviewers’ concerns that multiple parameter sets may be able to explain our results, so we have now performed a thorough investigation of the dependence of the results on specific parameter values (Supplementary Figure 3). These are now described in the manuscript (see also response to reviewer 1 point 1). The new analyses indicate that the results are robust and we believe it is reasonable to determine differential effects of CCh and noradrenaline based on these data.

In the numerical simulations of the manuscript (e.g. Figs 3, 4) specific sets of parameters for short-term plasticity were used, without taking into account their uncertainty. It remains unclear (at least to me) which parameter changes in the end controls the CCh induced change of the E-I ratio in Fig. 3E or back-propagating APs in Fig. 4.

This is somewhat related to the previous point. We now investigate the uncertainty associated with the specific choice of parameter values related to the effects of the two neuromodulators by exploring the space spanned by the model parameters in terms of their correlations. These show that although multiple parameter sets can in theory explain our results, if we apply the appropriate priors to constrain the parameter fitting process the confidence in our results is high. The critical parameter changes that control the CCh induced change in E-I ratio are the effect on feedforward inhibition where the increase in f0 reduces facilitation. For noradrenaline it is the reduction in inhibitory conductance. These changes are stated in the results section (lines 327-340).

Even though CCh strongly reduced inhibition, the simulated effects of the drug on E-I ratio (Fig. 3E) appear to me rather small. There is a an effect on the E-I ratio in Fig. 3E, in which the red region became slightly larger, but there is no fundamental change in the overall picture. Also the effects on back-propagating APs in Fig. 4CDE are not that striking, even though few dendrites may show a strong effect (Fig. 4B,C), which then could affect plasticity.

In Figure 3 we show the effects of CCh across a very large range of possible activity patterns. However, the physiologically relevant patterns that might drive formation of ensembles are a smaller subset and it is in these regions of the graphs that the effects of CCh are most dramatic. This is highlighted by the changes in spiking shown in Figure 4. In the examples shown, the transition from no spiking to spiking caused by CCh (or noradrenaline) occurs in exactly the activity range thought to be most physiologically relevant. The resulting change in Ca^2+^ signalling is dramatic and likely crucial for induction of synaptic plasticity. We make this point in the results section (lines 464-479).

I found that the two sentences on line 436 exaggerate the effects of CCh and NA: ``Our simulations indicate that disinhibition of the feed-forward mossy fiber pathway by acetylcholine or noradrenaline is a requirement for reliable induction of synaptic plasticity between CA3 pyramidal cells when CA3 ensembles are activated by mossy fiber inputs. Furthermore, acetylcholine reduces the number of stimuli within a burst required to generate substantial dendritic calcium whereas noradrenaline only facilitated the back-propagation of action potentials once they were initiated and is therefore only important for calcium signalling driven by longer bursts with more stimuli.'' Again, there are strong effects in some examples dendrites (Fig. 4B), but on average the effect is seems to be weak (Fig. 4 DE).

We believe the effect on a major portion of the dendrites is sufficiently large to likely be the difference between induction of plasticity and no plasticity. Consider, for example, the relative increase of ~50% in the probability of back-propagating action potentials reaching oblique dendrites shown in Figure 4H. However, we agree that this conclusion needs to be placed in the context of the likely physiological activity patterns and we have now adjusted the text to reflect this (lines 472).

Starting with Figure 5, the manuscript becomes more speculative and merely illustrates various scenarios related to synaptic plasticity. Now, very different kinds of numerical simulations of different sets of models are used, and these models depend on multiple (often unconstrained, ad-hoc chosen) parameters. Figs. 5, 7, and 8 deal with fully connected networks of excitatory ind inhibitory point neurons, spike timing dependent plasticity rules including scaling rules ``to maintain constant overall spiking dynamics'', and short-term plasticity models as in Fig. 2. However, the firing statistics are now different from what was used earlier.

To explore the effects of acetylcholine and noradrenaline on the formation of ensembles of neurons in CA3 we needed to adopt a different model. The parameters used in the network spiking model are all taken from experimentally determined values (Brown and Randall 2009, Buhl et al 1996) and have previously been used to constrain similar models (Hummos et al 2014). In this model we assume that acetylcholine and noradrenaline enable the induction of synaptic plasticity within the CA3 recurrent network since this is what we found in the previous section of the manuscript and we use experimentally determined rules for the induction of synaptic plasticity (Mishra et al 2016) coupled with previously proposed rules for homeostatic scaling and inhibitory synaptic plasticity (Turrigiano and Nelson 2000; Vogels et al 2011). As the reviewer points out, the latter is required in order to maintain constant overall spiking dynamics and is a common feature of all network models that incorporate plasticity. We have now reworded this section (lines 493-498) to clarify the approach adopted.

In the simulations in Fig. 5, mossy fiber input was compared to perforant path input. But very specific assumptions on firing rates of PP inputs (line 479) were made (based on data from the pre grid-cell era). Findings in Fig. 5 probably depend critically on many details of the model setup and the chosen parameters, which was not investigated. Yet another firing statistics for mossy fibers was used in Fig. 5C (winner take all process with adaptation). What was the motivation for this set of simulations? Overall, Fig. 5C (text, figure, and caption) was puzzling to me, and I think Fig. 5C is not well enough connected to other parts of the manuscript.

The firing rates for PP inputs were modelled based on experimentally observed firing patterns from multiple laboratories and we now include relevant references for grid cell firing patterns. Entorhinal neuron firing patterns are heterogeneous (see eg Mizuseki and Buzsaki 2013) and we have chosen distributions that span a reasonable sample of the observed range.

The motivation for the simulations of Figure 5C were to test the assumption that single granule cell inputs to CA3 pyramidal neurons are capable of forming ensembles and if so whether this depends on the observed short-term facilitation features of mossy fibers. We believe this is an interesting and important point to test within the model and we prefer to keep it in the manuscript. We now provide a clear exposition for the rationale within this section (lines 544-576).

Fig. 6 illustrates an experimental result. Prince et al. confirmed earlier results from other groups: CCh caused a ~50% depression of recurrent CA3-CA3 excitatory synaptic transmission and there is an increase in paired pulse ratio (PPR). Furthermore, CCh increased input resistance (but this result seems to be not significant: see Fig. 6D, right) and depolarized CA3 pyramidal cells. New (to my knowledge) is that NA had no (significant) effect on CA3 neurons and the recurrent synapses.

This is correct. The effects of acetylcholine in this regard have been reported previously as cited in the manuscript. The surprising result is the lack of effect of noradrenaline on CA3 network as highlighted in the discussion. It is important to include the effect of CCh as a comparison and to confirm these previous findings in out system.

In Fig. 6, are the stimulations used to evoke responses also ``minimal'' as for the mossy fibers? Or are the shown responses the sum of inputs from many active synapses? The EPSC amplitudes (200 pA in Fig. 6A) are comparable in magnitude to mossy fiber EPSCs in

Fig. 1, even though I thought mossy fiber synapses are much stronger than recurrent synapses in CA3. Please clarify.

The stimulation settings for activation of CA3 recurrent collaterals are not minimal and we expect that multiple axons and therefore synapses are activated by these stimulations. We now make this clear in the methods (lines 932-933).

Figure 7 illustrates with simulations ensemble formation in CA3. For the simulations in Fig. 7, NA was assumed to ``enable synaptic plasticity between CA3 pyramidal cells'' (line 575). Please add here the citations for experimental evidence on this. Was in Fig. 7 CCh assumed to NOT enable synaptic plasticity? Or was plasticity assumed to be the same for NA and CCh? I think this point should be made very clear and explicit here. This is important to be able to interpret the statement in line 589: ``Remarkably, when noradrenaline was switched for acetylcholine by applying the CA3 network effects, ensemble formation was rescued at the lower burst frequency.'' I find this surprising because earlier NA was proposed to control plasticity of these synapses. So, did the replacement of NA by CCh change (reduce) the plasticity of synapses? If yes, how was this implemented?

The reviewer highlights a point that was not made clearly in the manuscript. In the first part of the manuscript we show that both acetylcholine and noradrenaline can facilitate synaptic plasticity by enhancing E-I ratio in the mossy fibre pathway and in particular by reducing feedforward inhibition. This is what we were referring to on line 575. We then use these findings to assume that synaptic plasticity at CA3-CA3 synapses can occur in all the simulations shown in Figures 7 and 8 where noradrenaline or acetylcholine are present. What we show in these figures is that ensemble formation (rather than synaptic plasticity) is sensitive to the effects of acetylcholine on CA3 synaptic and network properties whereas noradrenaline has no observable effects. We now clarify the assumption for synaptic plasticity on line 615-617.

For the simulations shown in Fig. 7, to mimic the effects of CCh, the resting membrane potential for excitatory cells was depolarized by 5 mV to -70 mV, which was motivated by experiments (Fig. 6D). However, ``inhibitory cells were also depolarized to -63 mV'' (line 573). Why is this necessary? Is there experimental evidence on this effect?

The simulation of inhibitory neuron depolarisation was done to replicate observed experimental evidence (Lawrence et al 2006). This is now cited at this point (line 619).

Fig. 7B: How long were the simulations? (for comparison with Fig. 7C)

Our spiking networks were integrated for 400 seconds of simulated time. This is now stated on lines 636-637.

The main finding of Fig. 7 is summarized here ``...key factor [for ensemble formation] was the increase in cellular excitability, as removing the parameter changes to cellular excitability abolished the effect of acetylcholine but removing reductions in CA3-CA3 recurrent connections did not'' (line 602). Why were the changes induced by acetylcholine on the STP model parameters not important? Was this even investigated in Fig. 7? That these question were not addressed indicates to me a lack of coherence of the manuscript.

As described above, in Figures 7 and 8 we used the findings from Figures 3 and 4 to assume that noradrenaline and acetylcholine both enable synaptic plasticity. Therefore, we did not investigate the effects of acetylcholine and noradrenaline on STP with respect to ensemble formation. We emphasise again that the manuscript is organised into 2 parts. In the first we found that acetylcholine and noradrenaline facilitate the backpropagation of spikes into dendrites by disinhibition and in the second part we examine the effects of acetylcholine and noradrenaline on ensemble formation within the recurrent CA3 network.

I found the result in Fig. 7 not surprising at all given the fact that recurrent synapses (maximum conductance 0.5 nS in line 1077) should be much weaker than feedforward mossy fiber synapses, even though I could not find in the manuscript a number on the maximum conductance g of mossy fibers. My rough estimate from Fig. 1 (with current amplitudes in the range of 500 pA or more) and from the Supplementary Fig. 1 (assuming a driving force for excitatory currents in the range of 100 mV) is that maximum conductances of 500 pA / 100 mV = 5nS are easily achieved, and g should be even higher. The weak recurrent synapses are of minor importance for ensemble formation simply because they are weak.

The recurrent synapses are critical for ensemble formation and responsible for binding CA3 neurons together into the ensemble. The point made in Figure 7 is that reducing the strength of all the recurrent synapses by release of acetylcholine doesn’t have much effect on the speed at which ensembles are created whereas the increase in excitability caused by acetylcholine does. Conversely, in Figure 8 we show that the reduction in recurrent synapse strength allows ensembles to be created with greater overlap. It is important to show both to illustrate the roles played by the separable actions of acetylcholine. These may not be surprising to the reviewer, but we think they are still important to demonstrate.

More interesting in this respect are the results in Fig. 8: A reduction in strength of the (weak) CA3-CA3 recurrent synapse mainly determines the ability for acetylcholine to allow greater ensemble overlap. However I think this insight is not really new, and already Hasselmo 1999 explained in great detail that acetylcholine is could be involved in ``partially suppressing excitatory feedback connections and so facilitating encoding without interference from previously stored information''.

This is correct and we do confirm the results and conclusions found by Hasselmo in a series of papers in the 1990’s that we discuss and cite in the manuscript. The comparison with noradrenaline is novel and for the reasons we state in the manuscript show that the two neuromodulators have very different effects on the network coding properties.

In summary, in the simulations shown by Prince et al. the huge parameter space was not explored. Often, many specific parameter values were chosen arbitrarily, I think. The theory is therefore more like an illustration of how it could be rather than a framework from which solid, experimental testable predictions could be derived, and how these predictions quantitatively would depend on parameters.

We disagree with this assessment for all the reasons provided above. Principally, we hope that it is now clear how and why parameter values have been chosen. We also provide new analysis of our results using the STP model in Figure 2 based on an examination of the wider parameter space (see Supplementary Figure 3). Finally, we now include additional testable predictions as also requested by reviewer 1 (lines 834-863).

A limitation of the approach by Prince et al. is that they neglect the putative neuromodulation of direct inputs to dendrites of CA3 cells from entorhinal cortex (even though this perforant path was considered in simulations in Fig. 5). They fully neglect feedback from CA3 to DG via mossy cells, which might modulate granule cell activity for repeated stimulations. The authors could better justify why these features of hippocampal circuits are neglected.

The reviewer raises important components of the dentate gyrus-CA3 circuit but we do not believe these are relevant to our study for the following reasons. The perforant path input is not used in our model to generate ensembles. Whilst it is undoubtedly important for the activation of ensembles once they have been formed this is not the primary focus of our study. In Figures 7 and 8 we investigate the impact of acetylcholine and noradrenaline on ensemble formation driven by mossy fibre inputs. The feedback circuit from CA3 to DG via mossy cells is important for regulating mossy fibre activity patterns but this will already be incorporated into the granule cell activity patterns we use since these are based on those recorded in vivo.

MINOR ISSUES

line 251: delete extra ``a'' before ``produced''

Corrected.

line 317: replace ``increase in p0'' by ``increase in f0'' ?

Corrected.

line 323: ``increase in the facilitation incremental parameter tau_d.'' Do you mean here the ``depression incremental parameter''? Moreover, the text does not match results shown in Fig. 2F in which NA was indicated to significantly change only tau_a

Apologies, the star in Figure 2F was misplaced during rendering of the figures and should have indicated a change in tau d. This is the decay rate for the facilitation increments and is therefore correctly referred to in the text.

Fig. 3B,C. the EPSCs are plotted too small to be able to evaluate short-term plasticity. Please use a different current scale bar for EPSCs (maybe 100pA or lower).

We have now increased the size and hope this is now suitable.

Caption of Fig. 3 describes ``Pulse ratios for 2nd, 4th and 6th pulses'' whereas panels D and E figure shows ``n=2, n=6, n=10''

The title at the top of the panels shows Nth pulse ratios so we have now adjusted the sub panel figures to read N rather than n.

Caption of Fig. 5C: ``50 feed-forward inputs to a single granule cell'' . Do you mean ``... single pyramidal cell'' instead?

Thank you for pointing out this error. The legend is now changed to read pyramidal cell.

line 1079: I could not find the announced ``Table 1'' on network parameters

Table 1 is in the supplementary material.
