# Supplementary material for: Separable actions of acetylcholine and noradrenaline on neuronal ensemble formation in hippocampal CA3 circuits
Source: PLoS Comput Biol. 2021 Oct 1;17(10):e1009435. doi: 10.1371/journal.pcbi.1009435 (PMC8513881; doi:10.1371/journal.pcbi.1009435)
Supplement: Attachment — Submitted filename: Prince PLoS Comp Biol Rebuttal #2 - Luke v2.docx [file pcbi.1009435.s011.docx]

Response to reviewers’ comments (comments in black, responses in blue).

Many thanks for the revision of the manuscript, which helped a lot to better understand what was done. However, there are still many open issues:

Fig.1. In D and F, controls look very different, in particular the first pulses, which is much smaller in D. I think one should therefore no oversell the resulting extremely high nth pulse facilitation ratios in D because controls are so much larger in F (even though controls in D and F should not be significantly different... are they?).

Mossy fibre evoked responses are indeed highly variable due to the very low probability of release. This is particularly noticeable here when the stimulation intensity is reduced to minimal levels to activate very small numbers of axons and therefore release sites. There is a difference between the facilitation ratios for controls in D and F but as the reviewer suggests we do not draw any conclusions from these observations and they are accompanied by an explanation in the text. The key observation is made for within experiment changes in facilitation ratio due to application of carbachol or noradrenaline, which should be independent of initial facilitation ratio. This shows that carbachol causes a change in facilitation for IPSCs but not EPSCs whereas noradrenaline has no effect on mossy fibre transmission. These results are confirmed for irregular stimulation patterns in Figure 2.

Fig. 1: In E and F, a ``washout'' is mentioned in the caption but the time course is not shown in the corresponding panel. The reasons for not performing a washout experiment were explained in the revision. However, please delete ``and washout'' in the caption of E and F.

Thank you for picking this up. Mention of washout is now deleted.

In Fig. 2E, right panel, I think the star indicating significance should be above the dark red bar (for CCh) and not above ``Control''.

The reviewer is correct the star should be above the CCh bar. This is now corrected.

Line 322: Is ``facilitation incremental parameter'' the correct description of tau_d ? I thought it is the time constant of recovery of d from depression (according to Equations 5 and 13).

Thank you for picking this up. A better description at line 302 is depression recovery parameter which is now corrected.

Is the f^2 model (with parameters found there) used in subsequent models used to model EPSCs? Same question for the a-d-f model and IPSCs. Please state this explicitly somewhere in the manuscript to guide the reader. So far, I think, this is not the case. And even in Fig. 3, I am not sure what is used. It is said that in Fig. 3, ``THE model for short-term plasticity dynamics'' (line 359) was investigated when bursts are applied. However, I could not find an explicit note on which model was used (among the various models investigated in Fig. 2 E and F and discussed in the methods). And the models should differ for excitation and inhibition. What parameters values were used in Fig. 3? I think it is essential to summarize all used values (maybe in a table) and to make it very clear if (and then how) short-term plasticity was modeled here and in all following simulations in the manuscript. It seems that short-term plasticity is NOT explicitly modeled in some figures (7 and 8?), but this remained unclear to me.

The reviewer is correct that the f^2^ model is used for EPSCs and afd model for IPSCs in Figures 2 and 3 and we now make this explicit in the text (lines 287, 337). We also now provide in Table 1 (methods) all the parameter values used in the simulations in Figure 3 for control, noradrenaline and acetylcholine conditions. This is a useful addition to clarify the model parameters used and we thank the reviewer for this suggestion.

Short-term plasticity in the mossy fibre pathway is modelled in Figures 7 and 8 (line 560) but short-term plasticity at recurrent CA3-CA3 synapses is not. In addition, we assume that acetylcholine and noradrenaline both enable long-term plasticity at recurrent CA3 synapses as a result of the effects on short-term plasticity at mossy fibre inputs. This is stated in the paragraph at line 565.

In Equations 7-9, steady-state values at the beginning of a burst were derived. Why do these steady-state values not depend on the number of stimuli within in a burst and on the parameter Delta t_within? In the end, I failed to confirm Equations 7, 8, and 9. It would be good to give a hint how they were derived.

The steady-state values do not depend on delta t_within because it is assumed that by the time of the burst, the effect of the previous burst is negligible.

Equations 7, 8, and 9 can be derived from rearranging the iterative update rules, in particular equations 23, 24, and 26 as n-→ infinity.

Why is the symbol b in Equation 7 (and Equation 6) different from the symbol b_a in Equation 16?

Thank you for picking up this mistake – equations 6 and 7 have now been corrected – b now reads b_a

For Figure 3B it would be good to say (repeat) that EPSCs are red and IPSCs are blue.

Addition to figure legend made.

What is the ``Facilitation Ratio'' shown in Fig. 3C (top)? Which pulse number (in the burst) was compared to (I guess) the first one? Or is the ``Facilitation Ratio'' the steady-state amplitude of the first response in a burst compared to the baseline amplitude for an isolated pulse? Whatever, please explain the ``Facilitation Ratio'' somewhere close to this figure.

Was the ``paired pulse ratio'' in Fig. 3C (bottom) the ratio between amplitudes of response to first and second pulse in a burst? In steady state? If yes, please write this in the text or caption related to figure 3.

Frequency facilitation at the mossy fibre synapse is defined as the steady-state facilitation in response to low frequency stimulation and is distinct from the paired (or nth) pulse ratio comparing the 2^nd^ (or nth) pulse amplitude to the first in a high frequency burst. We now make this clear in the text (line 356).

Line 391: ``CCh dramatically reduced ... the initial IPSC amplitude... at all between and within burst intervals (Fig. 3B,D)'' This is not backed up by Figure 3B in which the first IPSCs appear only mildly reduced for 50s inter-burst intervals.

The wording of this sentence is now adjusted to more accurately reflect the results (line 370). The dramatic reduction in IPSC amplitudes is most prominent over the course of the burst and not for the initial response.

Line 430: Chamberland et al (2018) must be included in the list of references.

The reference is now included.

Fig4 B shows an example without back-propagating APs in control (and thus without dendritic Ca2+) even after 12 pulses. Why is then the largest value in Fig. 4D ``Control'' at around 8 pulses? Is the example in 4B not representative? If yes, please show a more representative case in B. Otherwise, is the emergence of a back-propagating APs defined in a different way? I think the text around Fig 4 lacks (but urgently needs) a clear definition of a back-propagating AP (threshold value for voltage or calcium in dendrite).

Back-propagating action potentials were defined as crossing a threshold membrane potential value of 0 mV in dendritic compartments, following a somatic action potential (crossing a threshold of 0 mV in somatic compartment). This is now stated in the text (line 414).

The values in Figure 4D only include instances where there was a back-propagating action potential in that dendrite. As can be seen in Figure 4E many of the dendrites had few instances of back propagating action potentials in control conditions. Therefore, the example traces shown in Figure 4B are representative, but these instances are not shown in Figure 4D. The figure legend for 4D is now adjusted to better describe the data shown.

Fig. 4E and F: unit ``millivolts'' of bAP amplitude missing on horizontal axis.

This omission is now rectified.

In Fig. 4F and G, the sum of probabilities indicated by the bars/dots is different from one (even though it should be one for correct normalization, I think). Please clarify. In Fig. 4F, if I assume that bars for Delta bAP > 40 mV(!) indicate the increased fraction of bAPs in the presence of ACh. But then this fraction is only ~3 percent (3 bars >0.5 percent), which I find pretty low. This number 0.03 does not at all match the one in Fig. 4H (~0.37 for gI/gE = 1)... there is an order of magnitude difference.

Thank you for pointing out this error. The issue was bar heights were not corrected for width (bar width = 5, sum of bar heights was 0.2) and the scale was therefore out by a factor 5. This has been corrected.

The values computed for 4H are for bAP amplitude > 40 mV not delta bAP > 40 mV, i.e., the sum of bars > 40 mV in 4F, not 4E.

Line 432: typo ``statrum'' -> ``stratum''

Line 434: reference before full stop?

Corrected.

line 452 ``the number of action potentials initiated during a train of stimuli did not [increase with noradrenaline]'' Do you mean ``... did not decrease'' or ``... were not reduced'' instead?

We meant to say that although the probability of action potentials increased, because of the marginal effects of noradrenaline the number did not increase and we now make this explicit (line 426).

Line 470 (sorry to bring this up again, but I am still not convinced): I think that the results shown up to Fig 4 (see also my remarks above on Fig. 4) do not support this sentence: ``Our simulations indicate that disinhibition of the feed-forward mossy fiber pathway by acetylcholine or noradrenaline are important for reliable induction of synaptic plasticity between CA3 pyramidal cells when CA3 ensembles are activated by mossy fiber inputs at physiologically relevant patterns.'' What was shown is that ACh might be able to MODULATE plasticity, and NA's effect is much weaker. I have not seen anything that justifies to say at that point ``reliable induction of synaptic plasticity'' because such plasticity between CA3 pyramidal cells was not yet addressed in the manuscript (only in Fig. 5).

This is a good point. We do not show evidence that acetylcholine enables reliable induction of plasticity, only infer that it will facilitate this. We then assume that this will enable plasticity induction in later simulations in Figures 7 and 8. We appreciate that it is important to make this absolutely clear and we have now adjusted the text accordingly (line 445).

Caption of Fig. 5: Replace ``Ppost'' by the symbol \rho_post (two times). What was the numerical value of \rho_max used in the simulations? I could not find it in the manuscript.

Replacement done. Rho_max was 10 Hz and this is now stated in the methods (line 1551).

Line 505: ``an ensemble was defined as being formed when all synapses between cells within the same ensemble had reached their maximum weight''. How was the maximum weight defined? What was the numerical value used in simulations? I guess that this weight is related to the symbol ``w'' in Equation 37 (Line 1145), which is different from the ``maximum synaptic conductance'' (with a misplaced tilde) in line 1147, which was fixed to 0.5 nS for excitatory synapses (line 1149). Puzzling is then the sentence in line 1172: ``Additionally, STDP was bounded between 0 and the maximum conductance of the synapse.''

The maximum conductance for excitatory synapses was 0.5 nS, and 1.0 nS for inhibitory synapses (stated on line 1069). The bounding means that regardless of any pre-post pair sequence or postynaptic firing rate, the synapse cannot exceed these values, i.e., synapses can only decrease in strength at these conductances. Therefore, STDP is bounded between 0 and the maximum conductance. Synaptic weight is equivalent to synaptic conductance and the relationship is now made clear in the methods (line 1069).

The scale and change of the weight w is defined by the symbol ``xi'' in Equation 39 (Line 1163). What was its value? I could not find it in the manuscript. And the vertical axes in Fig. 5B lack a scale for weights (there are just undefined w_EE and w with a tilde).

ξ = 0.02 and this is now stated in the text (line 1104).

W_EE and W both with tildes indicate the maximum weight, hence the scale is present relative to the maximum weight. Since excitatory and inhibitory synapses had different maximum weights, it made more sense to show them on this normalized scale.

It could well be that an even deeper dive into the manuscript (eg when one tries to reproduce the results in detail) uncovers more parameter values that were not defined. I therefore recomment to generate a new table that summarizes all the parameters used and states which values were used in the various simuluations shown in the figures.

This is a good idea and we have now created a new Table 1 for the parameters used in the Tsodyks-Markram model and moved Supplementary Table 1 (that had parameters used in the CA3 network simulations) into the main text as Table 2. These can both now be found in the methods section. We hope that all other parameters are also included in the methods section at the relevant points.

Line 537: I cannot agree with the following conclusion drawn from the simulations shown in Fig. 5A and B: ``These results show that mossy fiber-like sparse inputs and feedback inhibition within the CA3 recurrent network are important for rapid and stable formation of CA3 ensembles.'' The simulations In Fig 5B just have shown examples for very special settings of many many parameters that define the mossy fiber and perforant path inputs, the short-term plasticity, the neurons models (pyramids and interneurons), the spike-timing dependent plasticity, and the CA3 connectivity. Robustness of this conclusion to (even small) variations of any of these parameters was not shown. But this would be required to back up such a strong statement as cited above.

We now qualify the statement by indicating that these conclusions apply specifically to our model (line 540).

Given that Fig 5 A and B has already added an enormous amount of complexity of a network model of CA3, it is terrifying (to me) to see the extended setup for Fig 5C ``using this model'' (beginning of line 541) (Note that it was unclear to me which model exactly was used, for example what the values of the weights in the recurrent CA3 network were and if STDP in the recurrent weights was still on... which created a strong distrust in some claims).

In Fig. 5C, a single CA3 cell was considered, but a granule cell network was studied, and a new model of the input (winner take all process by Fukai and Tanaka 1997 with 2 new parameters: scale h and separation sigma) was introduced that was different from the input model in Figure 5B (why?). Then, short-term plasticity of mossy fiber synapses was turned off for comparison. Facilitation was found to give rise to ``a sharper transition between no transmission (efficacy = 0) and perfect transmission (efficacy = 1)'' (line 562). However, Fig. 5C also shows (not mentioned in manuscript) that almost perfect transmission (efficacy close to 1) is possible even without facilitation for a wide range of scales h and some intermediate separations sigma. I could not verify the statement in line 565: ``This indicates that short-term facilitation supports robust transmission of separated patterns, as weakly separated patterns can still be transmitted perfectly.'' If ``weakly separated'' means low sigma, then the blueish colors in the left graph of Fig. 5C for sigma=0.01 indicate efficacies very different from efficacy 1 (=perfect transmission). I therefore found the conclusion in line 572 unjustified: ``This supports the conclusion that ensemble formation is driven by single granule cell inputs to CA3 pyramidal cells in DG-CA3 circuits.''

Regarding again Figure 5C, I failed to decipher the sentence starting in line 549 (which defines the measure used to quantify the result): ``In the case of good transmission of a separated (winner) pattern, the postsynaptic cell should only spike (Fig. 5C; red trace) when the winning input is the only input with a non-zero weight i.e., once the winner has been selected (Fig. 5C; gray traces).'' The definition of the ``efficacy'' of transmission in Equation 10 (line 556) appears also mysterious to me: why using delta distributions there? If t is different from t_spike, the denominator is zero as well as the nominator. And if t = t_spike both are infinity. Finally, is the ``Winner efficacy'' plotted in Fig. 5C (scale bar) identical to the ``efficacy'' in equation 10?

On reflection we believe that the inclusion of Figure 5C is unnecessary to the core findings of the study and therefore it is best to remove it. We thought the findings were of interest, but they are somewhat speculative as the reviewer points out. The figure panel and references to it are now removed.

Line 588: ``increase in PPR (Fig. 6D;...'' not shown in D but in C

Line 591: part D of Fig. 6 not related to this

Lines 597 and 600: Fig. 6 EF does not exist

Thank you for pointing these errors out. They are now corrected.

Lines 602/3: noradrenaline effects not shown in suppl Fig. 1E,F

Text adjusted to remove reference to noradrenaline effects on spontaneous transmission.

Line 613: Unfortunately, I have pick on this again: ``...assumed that both neuromodulators enable synaptic plasticity at CA3-CA3 recurrent synapses in line with our previous results''. I understand that plasticity could be modulated (or enabled) by indirect effects of neuromodulators on the activity of neurons (and their dendrites), but this is not modeled in networks in Fig. 7 (with Izhikevich neurons, I guess). Fig. 4 showed a remarkable difference between ACh and NA in multicompartmental neuron models. So there should be difference between NA and CCh in enabling plasticity. And this requires a more precise statement on how strong NA and ACh may enable plasticity, and what the difference is between the two. I thought that this difference between ACh and NA is a major point of the manuscript. But all the sudden, both appear equally strong in enabling plasticity.

This is an interesting point that requires further explanation. As the reviewer has pointed out, we do not directly test the effects of acetylcholine and noradrenaline on induction of synaptic plasticity at recurrent CA3 synapses. Our simulations from Figure 4 indicate that both neuromodulators will facilitate back-propagating action potentials, and therefore plasticity, with acetylcholine having the greater effect. However, we are not able to quantify the degree of facilitation for each neuromodulator and therefore we have conservatively assumed that synaptic plasticity is capable of being engaged in the presence of either neuromodulator. We believe this is a reasonable assumption based on our simulations. The alternative is that plasticity can occur in the presence of acetylcholine but not in the presence of noradrenaline (or perhaps occurs much slower). This alternative is now discussed in the manuscript (line 751). We have also amended the description of the assumption to relate it more closely to the results of the simulations in Figure 4.

Fig. 7: How strong was the effect of NA compared to control? Was there even a difference to control? Why is ``control'' not show? What would be the difference to ``control'' in terms of the model parameters? Just the short-term plasticity parameters? Please explain! In your response letter it is mentioned that short-term plasticity was not used at all in Figures 7 and 8. I may have overlooked this in the manuscript, but I considered this possibility only after I read the response letter.

We apologise that this was not clear. In the absence of neuromodulators we assumed plasticity is inhibited and therefore no ensemble formation can occur. Therefore, the two conditions tested in Figures 7 and 8 are in the presence of noradrenaline or acetylcholine where each neuromodulator enables plasticity but have different effects on CA3 cellular and synaptic properties. It is this difference that we specifically wished to explore in these simulations. We now make this clear in the text (lines 544) and in Table 2.

The same question (How strong was the effect of NA compared to control?) applies to Fig. 8. Note that the related supplementary figures 4,5, 6 and 7 do not mention NA at all. And in the supplementary Table 1 the values for NA are identical to control values. Why, then should ``noradrenaline promote discriminative ensemble formation'' (line 688)?

Please see above. In the spiking network model the impact of noradrenaline compared to control is the ability for the network to undergo synaptic plasticity at recurrent connections. This enables ensemble formation in the presence of noradrenaline.

Line 623: I found the reference to Guzman et al 2016 misleading in the context of ``sizes of strongly connected assemblies'' because connectivity in Guzman et al was found to be close to one per cent (in contrast to the all-to-all connectivity in the model here) and assemblies had sizes of thousands of cells (in contrast to the 8 cells in the model here).

We agree this was misleading and we have now removed the statement that referred to the size of assemblies. We have also moved the Guzman reference to the statement where we say that biologically realistic connectivity patterns are in contrast sparse and larger than those in our simulations. For simplicity, we use small ensembles with all-to-all connectivity patterns which, although not necessarily biologically realistic do capture many of the features of a superconnected ensemble.

Line 1128: add unit ``mV'' after numerical value ``75'' of symbol ``v_r''. I guess, this symbol should have the same name as ``v_rest'' in Supplementary Table 1. This table shows essential model parameters, and should therefore not be ``supplementary material''. In the main text (line 613) it is even called ``Table 1''. In this table, also the control parameters should be given for easier comparison, for example to see that for NA parameters were not changed at all. I missed there an explanation/motivation why the parameters for ACh were changed in this particular way, and a link from the caption of the Table eg to Fig 6 would help, in particular when one needs to understand the choice of these values in the context of Fig. 5 and the related methods.

These changes are now made. The motivation for acetylcholine parameter changes are based on experimental findings summarised in Hummos et al 2014 (see line 1073).

Line 1004: Is mu_i defined somewhere (parameter value)?

*μ_1_* = 0.006 secs*, μ_2_* = 0.0025 secs. These values are now included (line 915).

Line 1011: What were the physical units of the parameter values? Seconds?

Seconds is correct and this omission is now corrected (line 915).

Line 1032: ``Equation 22'' -> `` Equation 23''

This is rectified.

Line 1155: Was the plasticity rule applied for every pre-post pair or only for next neighbors?

For every pre-post pair. This is now stated (line 1093).
