# Supplementary material for: Separable actions of acetylcholine and noradrenaline on neuronal ensemble formation in hippocampal CA3 circuits
Source: PLoS Comput Biol. 2021 Oct 1;17(10):e1009435. doi: 10.1371/journal.pcbi.1009435 (PMC8513881; doi:10.1371/journal.pcbi.1009435)
Supplement: Attachment — Submitted filename: Prince PLoS Comp Biol Rebuttal #3.docx [file pcbi.1009435.s012.docx]

Response to reviewers’ comments (comments in black, responses in blue).

Reviewer #2: I the second revision, all of my comments have been addressed. The authors should nevertheless check this:

The derivation of equations 7-9 was discussed in the revision. I now understand that delta_t_within could be neglected if it is small compared to delta_t_between. However, I still do not see why the number of spikes within a burst can be neglected. I could derive, for example, eq. 9 by assuming that there is one spike per burst. The explanation in the point-by-point response that "by the time of the burst, the effect of the previous burst is negligible" does not make sense to me because the anticipated steady-state values should depend on the structure of the bursts, which are characterized by several spikes.

The parameters determining the rate of decay of STP variables (tau_f, tau_d, tau_a) don't depend on the number of input spikes, and we assume that delta_t_within is negligible compared to delta_t_between. For the most part, tau_f, tau_d, tau_a are smaller than the range of delta_t_between values. As a result, we can assume that the effects of spikes on STP variables within the burst will have decayed by the start of the next burst. We acknowledge that these assumptions break down somewhat for smaller delta_t_between values, although for the types of spike statistics seen in mossy fibres (roughly 100 Hz bursts at rates of 0.1 Hz, Pernia-Andrade and Jonas 2014, [PMID 24333053](https://pubmed.ncbi.nlm.nih.gov/24333053/)), these assumptions should hold.

Equation number "26" does not exist, instead it has number "116".

This is now corrected.

Please check again the vertical scales in Figs 4F and G. In the point-by-point response it was said that a factor 5 was missing, but the figures were not updated.

Thank you for picking up this error. The correct version of the figure is now included.

The caption of Fig. 5 still shows "Ppost" instead of the symbol \rho_post

Capital rho looks very similar to capital P in this font but it is the symbol rho that is shown.

In Table 2, the bottom left entry shows just "~"

This is now corrected to show g_syn_
